# Exceptional Magnetocaloric Responses in a Gadolinium Silicate with Strongly Correlated Spin Disorder for Sub‐Kelvin Magnetic Cooling

**DOI:** 10.1002/advs.202306842

**Published:** 2024-02-14

**Authors:** Ziyu W. Yang, Jie Zhang, Bo Liu, Xiaoxiao Zhang, Dabiao Lu, Haoting Zhao, Maocai Pi, Hongzhi Cui, Yu‐Jia Zeng, Zhao Pan, Yao Shen, Shiliang Li, Youwen Long

**Affiliations:** ^1^ College of Civil and Transportation Engineering Key Laboratory of Optoelectronic Devices and Systems of Ministry of Education and Guangdong Province, College of Physics and Optoelectronic Engineering Shenzhen University Shenzhen 518060 China; ^2^ Beijing National Laboratory for Condensed Matter Physics Institute of Physics Chinese Academy of Sciences Beijing 100190 China; ^3^ School of Physical Sciences University of Chinese Academy of Sciences Beijing 100049 China; ^4^ Songshan Lake Materials Laboratory Dongguan Guangdong 523808 China

**Keywords:** cryogenic, gadolinium oxides, magnetocaloric effect, spin disorder, strong correlation

## Abstract

The development of magnetocaloric materials with a significantly enhanced volumetric cooling capability is highly desirable for the application of adiabatic demagnetization refrigerators in confined spatial environments. Here, the thermodynamic characteristics of a magnetically frustrated spin‐7/2 Gd_9.33_[SiO_4_]_6_O_2_ is presented, which exhibits strongly correlated spin disorder below ≈1.5 K. A quantitative model is proposed to describe the magnetization results by incorporating nearest‐neighbor Heisenberg antiferromagnetic and dipolar interactions. Remarkably, the recorded magnetocaloric responses are unprecedentedly large and applicable below 1.0 K. It is proposed that the *S* = 7/2 spin liquids serve as versatile platforms for investigating high‐performance magnetocaloric materials in the sub‐kelvin regime, particularly those exhibiting a superior cooling power per unit volume.

## Graphical abstract

It is hereby presented that the thermodynamic properties of a magnetically frustrated spin‐7/2 silicate exhibiting strongly correlated spin disorder, which provides promising platforms for investigating magnetocaloric materials with an enhanced volumetric cooling capacity, particularly those suitable for sub‐kelvin temperature applications.

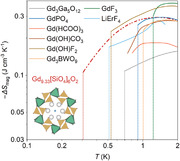

## 1 Introduction

Magnetic cooling, using adiabatic demagnetization refrigerators (ADRs) with an underlying magnetocaloric effect that describes the temperature change of a magnetic material subjected to field variations, presents a promising alternative to the prevailing cryogenic refrigeration techniques, such as the ^3^He sorption coolers and ^3^He‐^4^He dilution fridge.^[^ ^1^ ^]^ Besides alleviating concerns about the helium scarcity, ADRs offer notable advantages including enhanced reliability, compactness, simplicity, and more precise and stable temperature control capabilities, thus serving as attractive platforms for liquefying gases, cooling in space missions as well as the cutting‐edge quantum computing, even in cold atom experiments and realizing low‐temperature superfluid phases.^[^ ^2^ ^]^

In an ADR cycle, the cooling power *P* is calculated as the product of the working frequency *f* and the heat absorption capacity *Q*, *P* = *f*·*Q*.^[^ ^2c^ ^]^ Lifting the operating *f* can be achieved by minimizing the thermal resistances between the refrigerant and the heat sink/cold load, while increasing the factor *Q* is primarily determined by the working materials. Therefore, beyond the design of ADR architectures, the key stage of development should be an investigation of refrigerants that exhibit large magnetocaloric responses. The high‐performance magnetocaloric materials possess thermodynamic characteristics of: i) low lattice and electronic heat capacity to reduce the internal heat load; ii) low ordering temperature, which determines the minimum temperature achieved; iii) large magnetic moments; and iv) efficient thermal conductivity.^[^ ^2^, ^3^ ^]^ The spin‐only Gd^3+^ compounds, with large *J* = *S* = 7/2 and *g*_J_ = 2, then become rather appealing, as in the case of the benchmark refrigerant Gd_3_Ga_5_O_12_ (GGG), and the alternative dipolar antiferromagnet LiGdF_4_.^[^ ^2^, ^3^, ^4^ ^]^ Many other potential working materials, including the GdPO_4_,^[^ ^5^ ^]^ NaGdS_2_,^[^ ^6^ ^]^ Gd_2_SiO_5_,^[^ ^7^ ^]^ Sr_2_GdSbO_6_,^[^ ^8^ ^]^ Gd(HCOO)_3_,^[^ ^9^ ^]^ Gd(OH)CO_3_,^[^ ^10^ ^]^ GdF_3_^[^ ^11^ ^]^ and Gd(OH)F_2_,^[^ ^12^ ^]^ as well as the molecular {Gd_12_Na_6_} quadruple‐wheel are identified.^[^ ^13^ ^]^

In most cases, the volume of refrigerant dominates the magnet system and the associated shielding, which must be minimized during space missions, thereby posing a challenge in identifying magnetocaloric materials with a large volumetric cooling capability.^[^ ^2d^ ^]^ Generally, a higher magnetic ion density enables a greater cooling power density while also produces stronger interactions and consequently a higher ordering temperature. This is verified by the fact that only a few dense gadolinium compounds order in the deep sub‐kelvin regime, and persisting a pronounced volumetric magnetocaloric response down to the sub‐kelvin regime is hard to realize.^[^ ^5^, ^8^, ^9^, ^12^, ^14^ ^]^

The magnetic frustration and the emergence of a more certain collective paramagnet seem to offer a promising way out of this contradiction, wherein large degeneracy of the system ground state arises with suppressed long‐range ordering and enhanced ground state entropies.^[^ ^15^ ^]^ By balancing the exchange interaction strength over short‐range correlations, a 90% utilization of entropy storage capacity per mole Gd^3+^ ion is observed in frustrated *fcc* Ba_2_GdSbO_6_ and Sr_2_GdSbO_6_.^[^ ^8^ ^]^ The transition from the disordered spin liquid to the ordered up‐up‐down phases generates an attractive low‐field magnetic entropy change (−12 J kg^−1^ K^−1^) in the magnetically frustrated trillium lattice Na[Mn(HCOO)_3_].^[^ ^16^ ^]^ Furthermore, the unconventionally quantum critical point in the dipolar spin liquid KBaGd(BO_3_)_2_ permits an efficient magnetic cooling to well below the ordering temperature, which is identified as a Berezinskii‐Kosterlitz‐Thouless phase with emergent U(1) symmetry.^[^ ^17^ ^]^

However, it remains unanswered whether this strongly correlated spin disorder can contribute to the design of ADR materials with a substantial cooling capacity per unit volume. It is within this context that we present the magnetocaloric parameters of the oxyapatite Gd_9.33_[SiO_4_]_6_O_2_ (abbreviated to GSO henceforth), which exhibits correlated spin disorder from ≈1.5 K to at least 50 mK. Despite having a highly dense magnetic Gd^3+^ ion concentration (1.78 × 10^22^ cm^−3^), an exceptionally large magnetocaloric response is observed down to the sub‐kelvin regime. Our results indicate that the magnetic frustration within the spin liquid phase can enhance the volumetric magnetocaloric responses, allowing for possibilities to explore working magnetic refrigerants in systems with strongly correlated disorder, especially those featuring large *S* = 7/2 spins.

## 2 Results and Discussion

### 2.1 Crystallographic Structure

Polycrystalline GSO as well as its non‐magnetic analogue La_9.33_[SiO_4_]_6_O_2_ (La_9.33_) were synthesized by traditional solid‐state reaction. Powder X‐ray diffraction confirmed the space group P6_3_/*m* for the two compounds without any mixed phases of polymorphic disilicates or oxyorthosilicates (Figure S1, Supporting Information).

**Figure 1:**
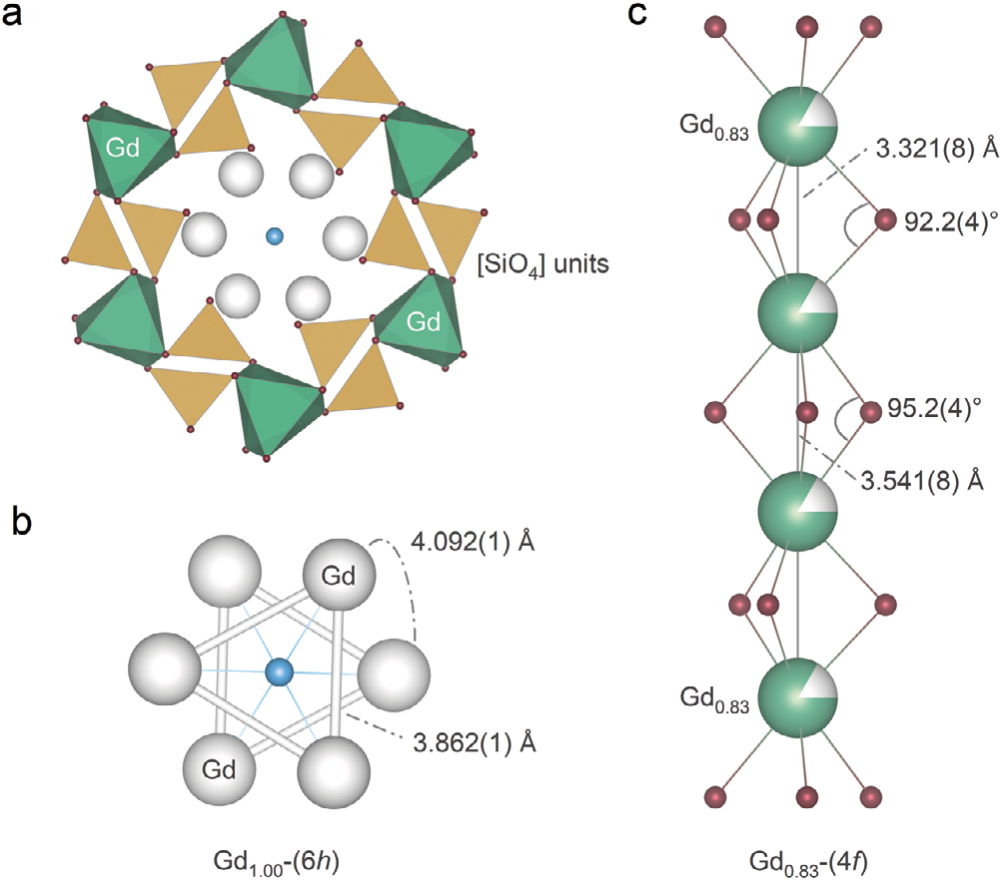
a,b) Perspective view on the crystallographic structure of GSO along the [001] direction, and c) the [010] direction. The 3 oxygen sites bonded to Gd_0.83_−(4*f*) and silicon are only shown in [SiO_4_] units for clarity. Blue balls indicate the special oxygen O4 located in channels at (0, 0, ¼) and (0, 0, ¾) along the 6_3_‐axis, while other oxygen sites are shown in magenta colour.

The GSO can be described as a cation deficient oxyapatitic structure, as determined by Smolin et al in 1969.^[^ ^18^ ^]^ In each unit cell, 2/3 cation holes are statistically distributed on the (4*f*) lattice sites (Gd_0.83_), which are surrounded by 9 oxygen atoms. All the 9 oxygens are silicon bonded (**Figure** **1**a), and the shortest Gd_0.83_−O bond is directed closely along [001] directions, to be of 2.305(9) Å with a Gd_0.83_−O−Gd_0.83_ angle of 92.2(4)°. The longer Gd_0.83_−O distance of 2.397(9) Å offers a larger Gd_0.83_−O−Gd_0.83_ angle, to be of 95.2(4)°, forming one‐dimensional chains with randomly distributed spins (Figure 1c).

Figure 1b shows the 7 oxygen atoms coordinated Gd_1.00_(6*h*) lattice site with an average Gd_1.00_−O distance of 2.320 Å. The “free” oxygen features the special position (0, 0, ¼) and (0, 0, ¾) along the 6_3_‐axis with an extremely short Gd_1.00_−O bond of 2.234(8) Å, forming stacked Gd_1.00_−Gd_1.00_ triangles. It should be noted that the shortest Gd_1.00_−Gd_0.83_ distance is 3.990(2) Å, marginally larger than the interchain distances of Gd_0.83_−(4*f*) sites, and comparable to the inter/intra bonds of Gd_1.00_−Gd_1.00_ triangles, which may bring three‐dimensional motif of correlation networks.

### 2.2 Magnetizations and Magnetic Modelling

**Figure 2:**
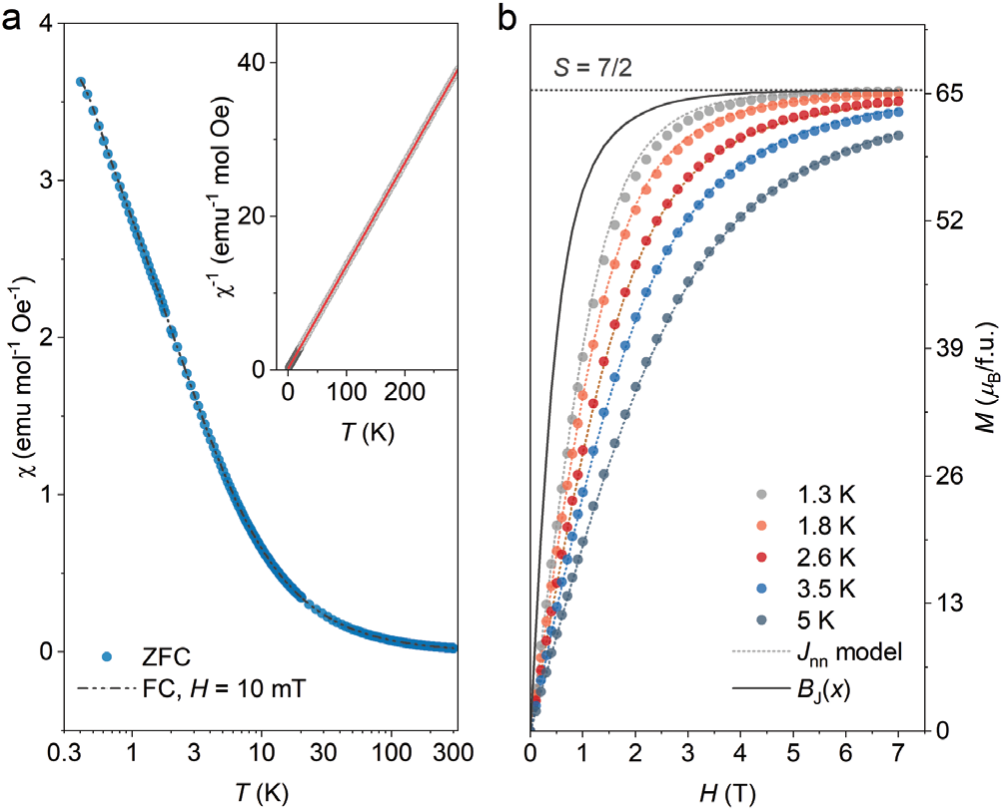
a) Constant field magnetic susceptibility *χ* down to 0.4 K of GSO in a 10 mT measuring field. Inset: Curie‐Weiss fit to the inverse magnetic susceptibility *χ* ^−1^ in the full temperature range to 300 K. b) Field‐dependent isothermal magnetization *M*(*H*) data reaching nearly 100% of the expected *g*_J_ *S*µ_B_ = 7 µ_B_ per Gd^3+^ ion polarized under *H* = 7 T. The corresponding mean‐field simulations with an isotropic interaction strength *J_nn_* = 0.16 K, *N_nn_* = 2, are shown as dotted lines. The solid line corresponds to the Brillouin‐like free spins *B*_J_(*x*) at *T* = 1.3 K.

Low‐field magnetic susceptibility and magnetization measured down to 400 mK are shown in **Figure** **2** and Figure S2 (Supporting Information), without any hint of long‐range ordering nor field‐induced phase transitions but correlated interactions below ≈9 K.

Data in the full temperature regime were modelled by Curie‐Weiss fits in the form *χ* = *C*/(*T*‐*Θ*_CW_) + *χ*_0_ (*χ*_0_ denotes the temperature‐independent contribution and accounts for a magnitude of 10^−5^ emu mol^−1^ Oe^−1^), giving an antiferromagnetic *Θ*_CW_ = −1.46 K with local moments of 7.73 µ_B_. The yielded magnetic moments are consistent with spin‐only Gd^3+^, $\mu_{\mathrm{eff}}=g_{J}\sqrt{S(S+1)}$ = 7.94 µ_B_, where the powder average *g*‐factor *g*_J_ = 2.

Although a magnetic field of *H* = 7 T was able to polarize Gd^3+^ moments up to the expected 7 µ_B_, the divergence from the Brillouin‐like free spins is obvious, especially under small *H* values (Figure 2b). The suppressed *M*(*H*) curve is consistent with the negative *Θ*_CW_ exchange parameter, indicating an effective coupling constant of *J_eff_* = 2.2 K if taking the number of nearest‐neighbour *N*_nn_ = 2. We then adopt a nearest‐neighbour Heisenberg model to characterize the bulk magnetic behaviour, by writing the spin Hamiltonian

(1) $$H=J_{1}\sum_{<i,j>}S_{i}\cdot S_{j}+H_{\mathrm{dip}}$$

of which *J*_1_ represents the Heisenberg exchange interactions between the nearest neighbour spin pairs, and H_dip_ denotes the magnetic dipolar interactions.^[^ ^7^, ^16^, ^19^ ^]^ Although the magnetic anisotropy is considered important in some Gd^3+^ clusters, we exclude the single‐ion anisotropy term for the eightfold degenerate spin multiplet of Gd^3+^ (*L* = 0, *S* = *J* = 7/2) here, following a similar approach employed in studies on garnet GGG and tripod kagome magnet Mg_2_Gd_3_Sb_3_O_14_.^[^ ^20^ ^]^ The relevant dipolar energy scale is *D*_dip_ = 0.65 K, using the crystallographic Gd^3+^…Gd^3+^ separation in triangle Gd_1.00_−(6*h*) chain, accounts for no more than 29% of the antiferromagnetic couplings.

By considering the Heisenberg exchange interaction *J*_nn_ as the governing parameter, which can be determined by incorporating an internal field *H*_i_,

(2) $$H_{i}\propto B_{J}\left[\frac{g_{J}J\mu_{B}\mu_{0}\left(H_{\mathrm{ext}}+H_{i}\right)}{k_{B}T}\right]$$

where *H*_ext_ represents the applied field, *k*_B_ denotes the Boltzmann constant, a fairly well description of the *M*(*H*) dependences is obtained, as shown in Figure 2b. The corresponding exchange field bounded moments are related to the degree of magnetic ordering, giving an isotropic antiferromagnetic interaction energy of *J*_nn_ *S* ^2^ = 1.96 K, which accurately captures the Curie‐Weiss fit.

### 2.3 Specific Heat and Entropy

**Figure 3:**
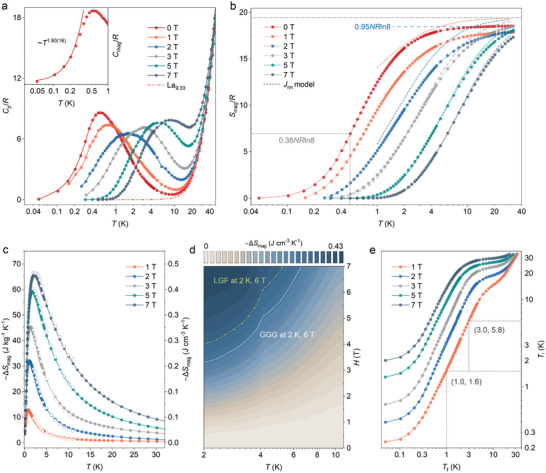
a) Specific heat *C*_p_ of GSO measured down to 50 mK under varying constant magnetic fields, and the non‐magnetic analogue La_9.33_ with an extrapolation to 0.5 K using the polynomial *AT* ^3^+*BT* ^5^ term. Inset: The low‐temperature portion of the zero field *C*_mag_(*T*) fitted to a *T^γ^* power law. b) Comparative magnetic entropy of experimental data and the mean‐field model. The resulting magnetic entropy *S*_mag_ approaches 95% of *NR*ln8 (*N* = 9.33) under zero field, and a corresponding 36% *NR*ln8 at the peak centred around *T* = 0.5 K. c) Magnetic entropy change *ΔS*_mag_ as a function of magnetic field and temperature determined from heat capacity (closed mark) and the isothermal magnetizations (open marks), and d) its comparison to the widely accepted ADR materials, the LGF and GGG under fields of *H* = 6 T at given temperature *T* = 2 K. e) Ideal adiabatic demagnetization and magnetization results under varied applied fields, starting point (3.0, 5.8) at *T*_i_ = 5.8 K and 1 T and reducing the field to 0 T resulting in a typical cooling to *T*_f_ = 3.0 K.

Specific heat measurements, *C*_p_, were collected down to 50 mK under zero‐field conditions and varied applied fields up to 7 T (**Figure** **3**a). The magnetic portion, *C*_mag_, was determined by subtracting the non‐magnetic analogue La_9.33_ as the lattice phonon contribution part (*C_L_*) that approaches zero below 2 K. By employing a single Debye model to fit the data, we obtained *Θ*_D_ = 374(5) K, as depicted in Figure S3 (Supporting Information). No sharp anomaly but a broad feature at around 0.5 K is observed, precluding the onset of any long‐range order, which is in consistent with the bulk magnetic susceptibility data.^[^ ^15a^ ^]^

The absence of a Néel transition gives rise to an empirical frustration parameter, *f* = |*Θ*_CW_|/50 mK ≈29, indicating significant frustration. It is noteworthy that the presence of a relatively large dipolar energy scale (*D*_dip_ = 0.65 K) breaks the rotational invariance and lifts the frustration, and circumvents the Mermin‐Wagner theorem.^[^ ^20a^ ^]^

The low‐temperature portion of the 0 T magnetic heat capacity *C*_mag_ was fitted to a power law *C*_mag_(*T*)≈*T^γ^*, gives a *γ* = 1.90(16), which does not follow the ∼ *T* ^3^ dependence that is usually observed in conventional ungapped antiferromagnetic magnon excitations, nor those with an anisotropy energy gap, *C*_mag_(*T*)≈exp(‐*ΔE*/*T*).^[^ ^21^ ^]^ Instead, the parameterized behaviour suggests the existence of unusual low‐energy excitations, similar to those with an apparent persistence of spin dynamics down to very low temperatures, such as the pyrochlore Gd_2_M_2_O_7_ (M = Ti, Sn),^[^ ^21^, ^22^ ^]^ the dipolar spin liquid KBaGd(BO_3_)_2_,^[^ ^17^, ^23^ ^]^ and the two‐dimensional Dirac spin liquid NaYbO_2_.^[^ ^24^ ^]^

Integrating the *C*_mag_/*T* up to 30 K gives a magnetic entropy *S*_mag_ reaching 95% of *NR*ln8, where *N* = 9.33 and *R* denotes the gas constant (Figure 3b). It should be noted that only 36% of the full entropy is released at *T* = 0.5 K, which contrasts with the classical Gd‐based antiferromagnets but resembles those commonly observed in triangular lattice of large spins, further arguing the differences from conventional antiferromagnetic spin waves.^[^ ^23a^ ^]^ The application of magnetic field induces an overall upward shift in the broad hump of specific heat *C*_p_, accompanied by a shift of entropy to higher temperatures, which can be attributed to the Schottky anomaly resulting from Zeeman splitting of the *J* = 7/2 ground multiplet.^[^ ^23^, ^25^ ^]^

We here used the incorporated *J*_nn_ parameter to capture essential features of the entropy curve, which provides a good quantitative description of the data taken under large fields and intermediate temperature regime (Figure 3b). The discrepancies between the model curve and the experimental results were adopted to modify the *S*_mag_‐*T* diagram, without disturbing the measured data at the lowest and the highest temperature.^[^ ^3b^ ^]^

### 2.4 Magnetocaloric Responses

**Figure 4:**
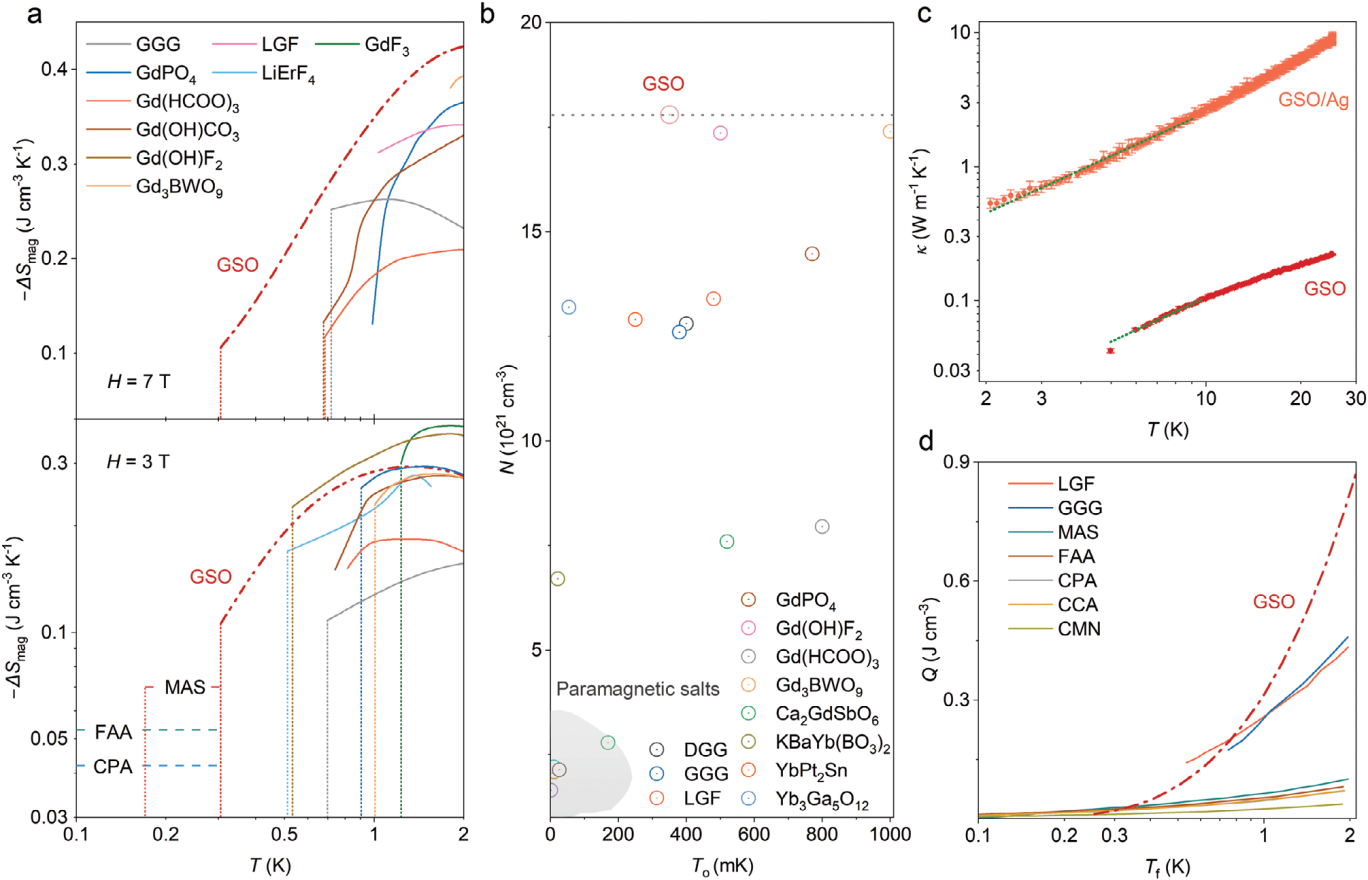
a) Magnetic entropy change of some representative ADR materials under external fields of 3 and 7 T with dotted drop lines corresponding to the phase‐transition temperature *T*_f_. Full entropy of the ground state is used for MAS, FAA, and CPA. Data extracted from Ref.[4, 5, 9, 10, 11, 12, 27] b) Parameters of ordering temperature and magnetic ion density for some common ADR materials. c) Thermal conductivity of GSO and GSO/Ag composites, along with a power‐law *αT^n^* fit. d) The heat absorption capability *Q* of various ADR materials, under *H*_i_/*T*_i_ = 3. Data extracted from Ref.[2c]. The abbreviations stand for: CMN‐Ce_2_Mg_3_·(NO_3_)_12_·24H_2_O, CPA‐CrK(SO_4_)_2_·12H_2_O, CCA‐CrCs(SO_4_)_2_·12H_2_O, FAA‐Fe(SO_4_)_2_NH_4_·12H_2_O, MAS‐Mn(SO_4_)_2_NH_4_·6H_2_O, and DGG‐Dy_3_Ga_5_O_12_.

The magnetocaloric effect can be related to the magnetizations via the thermodynamic Maxwell

(3) $$\Delta {S_{m}}_{\mathrm{ag}}\left(H,T\right)=\int_{0}^{H}\frac{\partial M(H^{\prime },T)}{\partial T}dH^{\prime }$$

or indirectly calculated from the heat capacity data.^[^ ^1^ ^]^ Figure 3c depicts the so‐obtained two sets of data, which agrees very well with each other. The determined isothermal magnetic entropy changes, −*ΔS*_mag_, are asymmetric, of which the extrema shift to higher temperatures with increased fields. The scaling analysis of the entropy curves above *T* = 2 K exhibits characteristics reminiscent of a second‐order transition, as illustrated in Figure S5 (Supporting Information). The maximum −*ΔS*_mag_ is 0.43 J cm^−3^ K^−1^ (66.6 J kg^−1^ K^−1^) at *T* = 2 K and *H* = 7 T, ≈85% of the maximum entropy storage capacity (9.33*R*ln8). If compared to the most two popular working magnetic refrigerants, GGG (Gd_3_Ga_5_O_12_) and LGF (LiGdF_4_), the GSO precedes in the full working temperature window both under high and low applied fields (Figure 3d and **Figure** **4**a).^[^ ^3^, ^4^, ^14^ ^]^

We further extend the comparisons to a range of potential ADR materials applicable in the sub‐kelvin regime, wherein GSO exhibits superior performance compared to most magnetic refrigerants, as illustrated in Figure 4a; Figure S4a (Supporting Information). More precisely, while GSO's magnetocaloric performance is only surpassed by the recently reported Gd(OH)F_2_ in terms of magnetic entropy change values, it boasts a lower terminal working temperature *T*_f_ and an overall larger adiabatic temperature change *T*_ad_.^[^ ^12^ ^]^ Further estimating the merit of relative cooling power, RCP, calculated as the product of the maximum of −*ΔS*_mag_ and its corresponding full width at half maximum, gives a fairly large value of 4.2 J cm^−3^ (644.3 J kg^−1^).

Figure 3e and Figure S4b (Supporting Information) depicts the temperature changes, *T*_ad_, associated with the adiabatic demagnetizations and magnetizations. Point (3.0, 5.8) denotes a *T*_ad_ = 2.8 K, if the GSO is at *T* = 5.8 K and magnetized in a field of 1 T, reducing the field to zero makes the temperature falls to *T* = 3.0. It is worth noting that the interpretation of adiabatic temperature changes becomes more intricate in practical devices, given the inherent challenge of achieving complete thermal insulation in refrigerant suspension systems. Further investigation into directly measuring *T*_ad_ would be intriguing, as exemplified by studies on molecular magnetic coolants [Gd_7_(OH)_6_(thmeH_2_)_5_(thmeH)(tpa)_6_(MeCN)_2_](NO_3_)_2_ and [{Gd(OAc)_3_(H_2_O)_2_}_2_]·4H_2_O, as well as on EuGd_2_O_4_.^[^ ^26^ ^]^

Figure 4b depicts the parameters of the ordering temperature *T*_O_ and the magnetic ion density *N* for some common ADR materials. In our cases, the GSO possesses almost the largest *N* but a relatively low *T*_O_ (data here taken as the quotient *C*_p_/*T*), benefiting from the magnetically frustrated configurations within the spin liquid regime.

The heat absorption capability *Q*(*T_f_*) in an ideal Carnot cycle can be estimated as

(4) $$Q\left(T_{f}\right)=\int \dot{Q}\left(T_{f}\right)\cdot dT$$

where $\dot{Q}\left(T_{f}\right)=T_{f}\cdot dS/dt$ represents the heat flux into the refrigerant.^[^ ^2b–d^ ^]^ The upper limit of *Q*(*T_f_*) is achieved at the point where the magnetic entropy change is the highest, and corresponds to the longest hold time. The GSO shows a salient volumetric *Q*(*T_f_*) as compared to GGG and LGF, as well as the widely used paramagnetic salts for a starting field and temperature of *H*_i_/*T*_i_ = 3 (Figure 4d), which renders the possible design of a more compacted ADR apparatus.^[^ ^2^, ^4^ ^]^ Additionally, the GSO exhibits a moderate contribution from lattice entropy, thereby enabling the attainment of a specific base temperature with a possible low ramping field.

If further considering the non‐thermodynamic properties, the GSO also excels in terms of availability and manufacturability, as well as in chemical and physical robustness. Challenges may arise in achieving optimal thermal conductivity performance, which can be effectively addressed by incorporating composites comprising highly conductive additives such as gold, silver, copper, or carbon nanotubes.^[^ ^2c,d^ ^]^ As illustrated in Figure 4c, the GSO shows a thermal conductivity of the magnitude of 10^−2^ W m^−1^ K^−1^ in the temperature range below 4 K, akin to the paramagnetic salt Gd_2_(SO_4_)_3_·8H_2_O,^[^ ^28^ ^]^ while incorporating the Ag powders (50 wt%) increased this value to 10^−1^ W m^−1^ K^−1^. An empirical fit with the expression *κ* = *αT^n^* gives a *n* value approaches 1, indicating strong scattering within the samples. Significantly enhanced thermal conductivity can be anticipated in single crystal forms, as evidenced by a series of rare earth oxides studies.^[^ ^29^ ^]^ Therefore, we conclude that GSO is a viable option for magnetic cooling in sub‐kelvin temperature, as well as the initial cooling stage to milli‐kelvin temperatures due to its capacity to cool down to the range of paramagnetic salts.

## 3 Conclusion

We have determined the magnetocaloric characteristics of the *S* = 7/2 spin liquid GSO through heat capacity and magnetization measurements, scaling analysis, as well as modelling of the internal exchange and dipolar interactions. The magnetically frustrated GSO exhibits an exceptional magnetocaloric effect down to the sub‐Kelvin regime, with a maximum isothermal magnetic entropy change of −*ΔS*_mag_ = 0.43 J cm^−3^ K^−1^ (66.6 J kg^−1^ K^−1^) at *T* = 2 K and *H* = 7 T, along with a corresponding maximum adiabatic temperature change *T*_ad_ = 23.8 K. Our results demonstrate that the strongly correlated spin disorder can be used to achieve an enhanced volumetric magnetocaloric response, even in systems consisting of dense large *S* = 7/2 spins, thereby open a promising platform for exploring superior working agents for ADR applications below 1 K.

## 4 Experimental Section

### Materials and Synthetic Procedures

Polycrystalline samples of ≈4 g were prepared using the traditional solid‐state method according to J. Felsche's protocol.^[^ ^30^ ^]^ Stoichiometric amounts of La_2_O_3_ (5N, Adamas‐beta, pre‐heated at 1173 K overnight), Gd_2_O_3_ (5N, Adamas‐beta, pre‐heated at 1173 K overnight), and SiO_2_ (3N, Energy Chemical, pre‐heated at 1173 K for 30 h) were thoroughly mixed in a mortar, and compacted into a pellet with a diameter of *φ*‐18 mm under a pressure of 15 Mpa for 30 min. Subsequently, the mixture was loaded into alumina crucibles with lid and annealed at 1573 K in a muffle furnace for 24 h under a flowing air atmosphere. After cooling down to room temperature, the samples were subjected to a repeated process of crush‐pellet‐anneal at 1573 K for 24 h twice until single‐phase samples were obtained.

### Magnetic and Calorimetric Measurements

The dc magnetic susceptibility and magnetization were measured as a function of the applied field (0 to 7 T) and temperature (0.4 to 300 K) using a Quantum Design SQUID magnetometer (MPMS‐III) equipped with a ^3^He‐refrigerator insert. Specific heat measurements were conducted on a Quantum Design PPMS (PPMS‐9) at various applied fields of 0, 1, 2, 3, 5, and 7 T down to temperatures as low as 50 mK using a ^3^He−^4^He dilution refrigerator. The thermal conductivity was measured using a P670 Thermal Transport System (TTO, Quantum Design) within the temperature range of 2–25 K. Polycrystalline samples with particle sizes smaller than 100 µm were compressed into cylindrical shapes (φ‐4 mm × 10 mm) under a pressure of 30 MPa. Silver powders used were finely powdered to achieve particle sizes below 5 µm. The final density was estimated to exceed 80% of the solid density.

## Conflict of Interest

The authors declare no conflict of interest.

## Supporting information

Supporting Information

## Data Availability

The data that support the findings of this study are available from the corresponding author upon reasonable request.
