# Supplementary material for: Exceptional Magnetocaloric Responses in a Gadolinium Silicate with Strongly Correlated Spin Disorder for Sub‐Kelvin Magnetic Cooling
Source: Adv Sci (Weinh). 2024 Feb 14;11(16):2306842. doi: 10.1002/advs.202306842 (PMC11040381; doi:10.1002/advs.202306842)
Supplement: Supplementary file 1 — Supporting Information [file ADVS-11-2306842-s001.pdf]

ADVANCED SCIENCE
Open Access

Supporting Information

for *Adv. Sci.*, DOI 10.1002/advs.202306842

Exceptional Magnetocaloric Responses in a Gadolinium Silicate with Strongly Correlated Spin Disorder for Sub-Kelvin Magnetic Cooling

Ziyu W. Yang, Jie Zhang, Bo Liu, Xiaoxiao Zhang, Dabiao Lu, Haoting Zhao, Maocai Pi, Hongzhi Cui, Yu-Jia Zeng*, Zhao Pan, Yao Shen, Shiliang Li and Youwen Long**

Supporting Information

Exceptional Magnetocaloric Responses in a Gadolinium Silicate with Strongly Correlated Spin Disorder for Sub-Kelvin Magnetic Cooling

Ziyu W. Yang, Jie Zhang, Bo Liu, Xiaoxiao Zhang, Dabiao Lu, Haoting Zhao, Maocai Pi, Hongzhi Cui, Yu-Jia Zeng*, Zhao Pan, Yao Shen, Shiliang Li, and Youwen Long**

[1] *College of Civil and Transportation Engineering, College of Physics and Optoelectronic Engineering, Shenzhen University, Shenzhen 518060, China.*

[2] *Beijing National Laboratory for Condensed Matter Physics, Institute of Physics, Chinese Academy of Sciences, Beijing 100190, China.*

[3] *School of Physical Sciences, University of Chinese Academy of Sciences, Beijing 100049, China.*

[4] *Songshan Lake Materials Laboratory, Dongguan 523808, Guangdong, China.*

I. Rietveld refinement

Powder X-ray diffraction patterns (PXRD) were collected at room temperature on a Huber diffraction and positioning equipment with monochromatic Cu Kα radiation. Rietveld refinements with the pseudo-Voigt function were performed within the GSAS package (EXPGUI), of which the Chebyshev polynomial of 10 terms was used to model the backgrounds[1, 2].

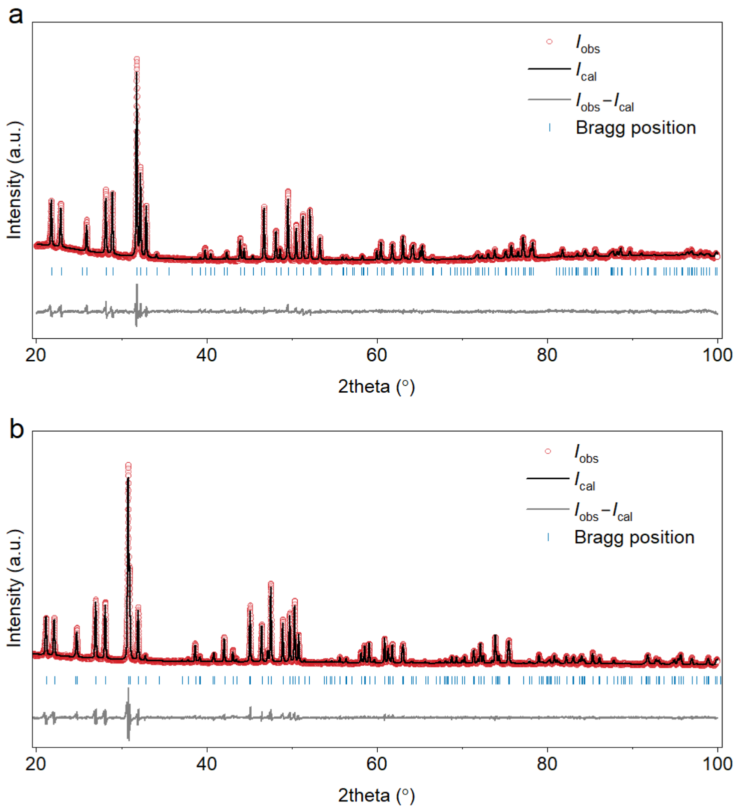

Figure S1. Observed and simulated diffraction patterns for GSO (a) and $La_{9.33}$ (b), along with the differences in Obs – Cal and the Bragg positions (vertical bars).

The PXRD patterns confirm the formation of single-phase GSO polycrystalline powders with all the observed reflections indexed into the $P6_3/m$ (176) space group. The derived structural parameters are $a = b = 9.4073(3)$ Å, $c = 6.8615(2)$ Å and $\alpha = \beta = 90°$, $\gamma = 120°$, with a calculated density $\rho = 6.4817$ g cm^{-3}. The goodness of fit χ^2 is 4.405, with final reliability indices of wRp/Rp = 0.0232/0.0164 = 1.41. The refined positional and thermal parameters and selective bond lengths and angles are listed in Table S1-2. Refinement of the experimental $La_{9.33}Si_6O_{26}$ pattern gives wRp/Rp = 0.0462/0.0309 = 1.50, and a goodness of fit χ^2 = 4.270.

Table S1. Crystallographic parameters and the equivalent isotropic displacement parameters of GSO.

Atom	Wyck.	S.O.F.	*x/a*	*y/b*	*z/c*	*U* [Å^2]
Gd1	6h	1	0.24075(17)	0.23313(16)	3/4	0.0059
Gd2	4f	0.8333	1/3	2/3	0.0080(5)	0.0059
Si1	6h	1	0.4065(6)	0.3787(6)	1/4	0.0007
O1	6h	1	0.3182(13)	0.4897(12)	1/4	0.0015
O2	6h	1	0.5959(13)	0.4585(12)	1/4	0.0015
O3	12i	1	0.3425(7)	0.2500(7)	0.0692(8)	0.0015
O4	2a	1	0	0	1/4	0.0015

Table S2. Selective bond lengths and bond angles of GSO.

Bond lengths			Bond angles	
From	To	d [Å]	Angle	Degrees
Gd2	Gd2	3.32097	Gd1_O1_Gd2	106.12(32)
Gd2	Gd2	3.54053	Gd1_O1_Gd2	106.14(32)
Gd1	Gd1	3.86217	Gd1_O1_Si1	97.0(5)
Gd2	Gd1	3.99032	Gd2_O1_Gd2	92.2(4)
Gd1	Gd1	4.09172	Gd2_O1_Si1	126.64(32)
Gd2	Gd1	4.11991	Gd2_O1_Si1	126.62(32)
Gd1	O4	2.22983	Gd1_O3_Gd1	116.63(25)
Gd2	O1	2.30477	Gd1_O3_Si1	138.1(4)
Gd1	O3	2.36356	Gd1_O3_Si1	99.61(31)
Gd2	O2	2.39736	Gd1_O4_Gd1	120.000(0)
Gd1	O3	2.44488	Gd1_O4_Gd1	120.000(0)
Gd1	O2	2.51375	Gd1_O4_Gd1	120.000(0)
Gd1	O1	2.67929		
Gd2	O3	2.79359		
Gd2	O3	2.79359		
Gd1	O3	3.34617		
Gd1	O1	3.63251		
Gd2	O3	3.9857		
Gd1	O2	3.98868		

II. Magnetic modeling

The free Heisenberg spin model that describes the bulk magnetization M, under an applied field of H and temperature T, takes the form

$$M = M_0 B_J(x) \tag{1}$$

of which the saturation magnetization $M_0 = Jg_J\mu_B$, $x = Jg_J\mu_B\mu_0 H/k_B T$, and $B_J(x)$ represents the Brillouin function,

$$B_J(x) = \frac{1}{2J}\left[(2J+1)\coth\frac{2J+1}{2J}x - \coth\frac{x}{2J}\right] \tag{2}$$

where $g_J = 2$ is the spectroscopic splitting factor for Gd^{3+} ions, $J = S = 7/2$ denotes the electronic spin, μ_B is the Bohr magneton and k_B is the Boltzmann constant.

The dipolar energy scale, D_{nn}, is estimated by taking the form,

$$D_{nn} = \frac{\mu_0 \mu_{eff}^2}{4\pi R_{nn}^3 k_B} \tag{3}$$

of which the effective magnetic moments, $\mu_{eff} = g_J(J^2+J)^{1/2}$, R_{nn} denotes the nn distance determined by the Rietveld refinements.

The effective exchange constant can be related to the Weiss temperature Θ_W via a standard mean-field estimate, by

$$J_{ex} = -\frac{3\Theta_w}{N_{nn} S(S+1)} \tag{4}$$

where J_{ex} is the exchange constant and N_{nn} represents the number of nearest neighbors for a single Gd^{3+}. We here adopted a crude molecular model to characterize the *nn* exchange, J_{nn}, by assuming that an internal field H_i is generated with the partial ordering of spin-7/2 Gd^{3+} ions[3-6]. The total effective field acting on magnetic ions then reads as

$$H_{tot} = H_{ext} + H_i + H_d \quad (5)$$

where H_{ext} and H_d represent the applied field and the dipolar field, H_i is proportional to the degree of order in the system. This approach is justified in the case of the ensembled isotropic Gd^{3+} spins (the contribution of orbital momentum L is zero) and in the analyzed paramagnetic regime where quantum fluctuations can be neglected. Considering the exchange part of the Hamiltonian only, we get

$$\mathrm{H}_J^i = J\sum_{i=1}^{N_{nn}} S_i S_j = \frac{1}{g_J^2 \mu_B^2} J \sum_{i=1}^{N_{nn}} \mu_i \mu_j \quad (6)$$

of which the average values are

$$H_i = \frac{N_{nn} J_{nn}}{g_J^2 \mu_B^2} M \quad (7)$$

By finding the root of the function

$$M = g_J J B_J (|H_{tot}|, T) \quad (8)$$

we then get the exchange parameter J_{nn}.

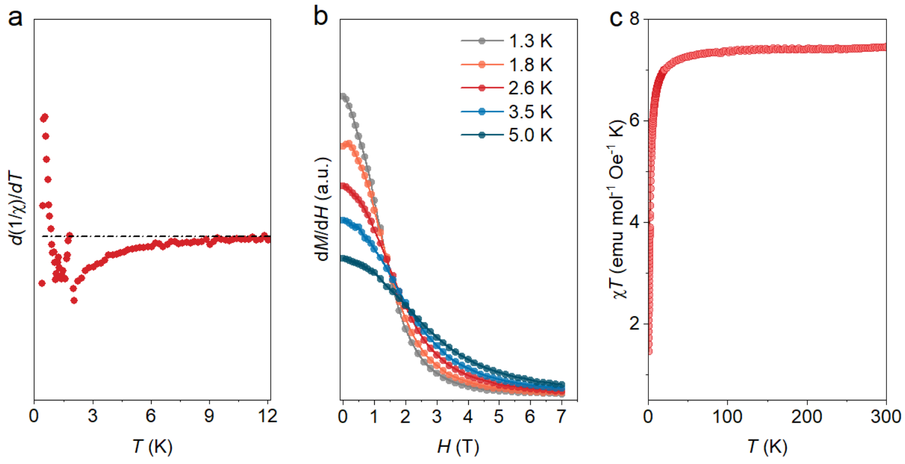

Figure S2. (a) The derivative of $1/\chi(T)$ (close marks), and the corresponding derivative of the Curie-Weiss fit (dash line). (b) The derivative of the field-dependent magnetization, dM/dH, down to 1.3 K. (c) The dc susceptiobility in ther form of χT *vs. T*.

III. Analysis of heat capacity and magnetic entropy

According to the thermodynamic Maxwell relation, the isothermal magnetic entropy change can be related to the magnetization dependences by

$$\left(\frac{\partial S}{\partial H}\right)_T = \left(\frac{\partial M}{\partial T}\right)_H \quad (9)$$

where the calculation is numerically approximated by

$$\Delta S_{\mathrm{mag}} \approx \frac{1}{\Delta T}\left[\int_0^H M(T+\Delta T, H)\,dH - \int_0^H M(T,H)\,dH\right] \quad (10)$$

using the magnetization data collected at discrete temperature T and field H intervals. Additionally, the numerical calculation can be processed from heat capacity data, by

$$\Delta S_{mag} = \int_0^T \frac{C_{mag}(T,H)}{T} dT - S_{mag}(T,0) \tag{11}$$

where $S_{mag}(T, 0)$ denotes the zero-field magnetic entropy, and $C_{mag}(T, H) = C_p(T, H) - C_L$ represents magnetic heat capacity. The low-temperature lattice contributions C_L (0.05 K < T < 3 K) is determined by fitting and extrapolating the heat capacity data of $La_{9.33}$ with a single Debye model,

$$C_L = \frac{9nN_A k_B T^3}{\Theta_D^3} \int_0^{\Theta_D/T} \frac{x^4 e^x}{(e^x - 1)^2} dx \tag{12}$$

where N_A denotes the Avogadro constant, Θ_D denotes the Debye temperature, and n is the number of atoms per chemical formula, respectively.

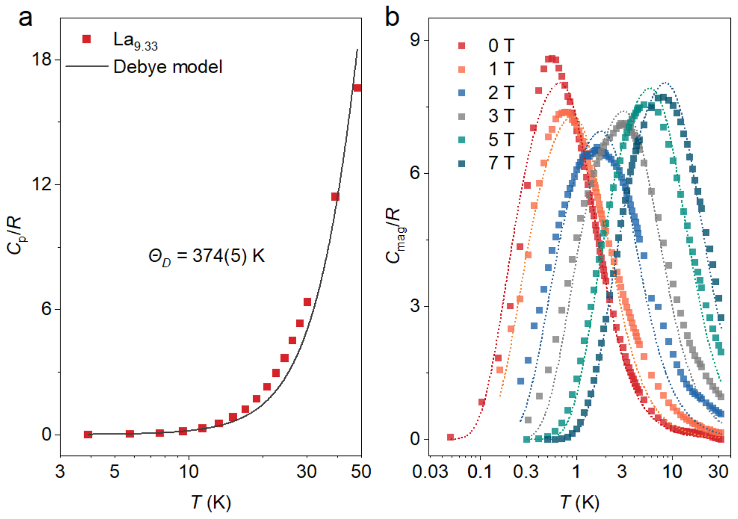

Figure S3. (a) Heat capacity of $La_{9.33}$ and the single Debye fit. (b) Temperature dependences of magnetic heat capacity C_{mag} under different magnetic fields. The dashed line represents a two-level Schottky fitting.

We then adopted a mean-field approach, corresponding to the J_{nn} approximation, to bring the calculated results into closer agreement with experimental values. The model here starts from a paramagnetic phase, where the magnetic entropy S_{mag} is related to the partition function Z by

$$\frac{S_{mag}}{R} = \frac{d(T \ln Z)}{dT} \tag{13}$$

in which case the partition function Z takes the form,

$$Z = \sum_{I=-J}^{I=J} \exp \frac{IM_J(H + H_i)}{Jk_B T} \tag{14}$$

where $M_J = g_J \mu_B J$ represents the magnetic moment of one atom. Summation of Eq. (13-14) gives the magnetic entropy S_{mag} per mole as

$$\frac{S_{mag}}{R} = \ln \frac{\sinh[(2J+1)x/2J]}{\sinh(x/2J)} - xB_J(x) \tag{15}$$

where $x = g_J\mu_B S(H+H_i)/k_B T$. The S_{mag}-T phase diagram was thus modified considering the discrepancy between the calculated data and the model results.

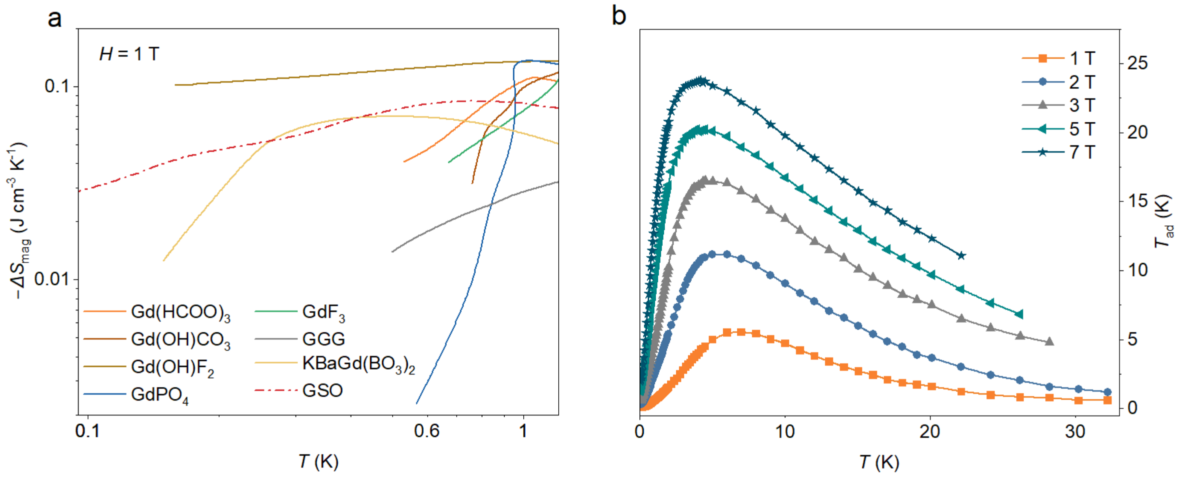

Figure S4. (a) Magnetic entropy change of some representative ADR materials under external fields of 1 T. (b) Adiabatic temperature change, T_{ad}, under varied constant fields.

IV. Scaling analysis

The order of magnetic phase transitions can be determined by assessing the universality of the magnetocaloric responses[7-11]. A single master curve (Figure S5) is constructed by normalizing the $-\Delta S_{mag}(H, T)$ datasets phenomenologically with respect to the corresponding maximum by $\Delta S_{mag}(H, T)/\Delta S_{max}$, and rescaling the temperature axis to

$$T_{nor} = (T - T_{max}) / (T_{ref} - T_{max}) \tag{16}$$

of which T_{max} represents the temperature of $-\Delta S_{max}$, and T_{ref} corresponds to the reference temperature. Experientially, the T_{ref} here was defined by the temperature corresponding to the half maximum of the peak values of the magnetic entropy change curves, $\Delta S_{mag}(H, T_{ref}) = 1/2\Delta S_{max}$[7-11].

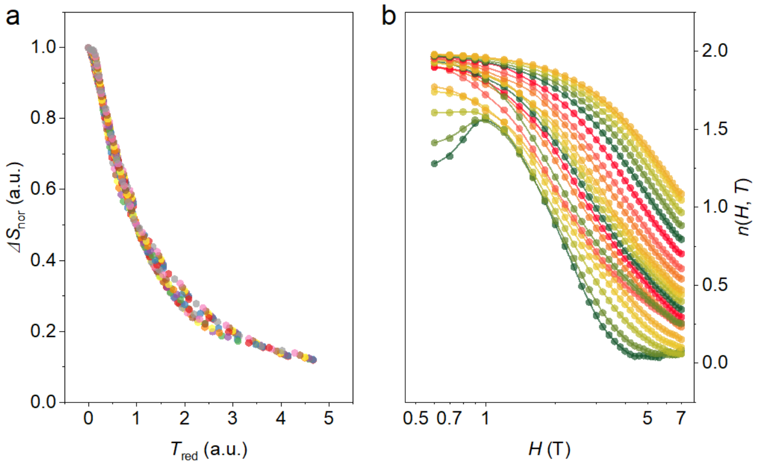

Figure S5. (a) The phenomenological universal curves derived from a total of 36 applied fields. (b) The field dependences of the exponent $n(H, T)$ of isothermal curves in the temperature range of 0.4– 11K.

A quantitative criterion is also adopted by further expanding the power law to the general field variance of the magnetic entropy datasets, $-\Delta S_{\mathrm{mag}}(H, T) \propto H^n$, in the form of

$$n(H,T) = \frac{d \ln |\Delta S_{mag}|}{d \ln H} \tag{17}$$

A scaling nature of the trend of $n(H, T) \rightarrow 2$ is identified, as illustrated in Figure S5, which resembles those of the second-order thermomagnetic phase transitions and in accordance with the universal analysis[10, 12]. It should be noted that the regions of low applied fields ($H < 0.5$ T) were omitted for possibilities of 'multi-domain' state[10].

References

[1] H. Rietveld, A profile refinement method for nuclear and magnetic structures, J. Appl. Crystallogr. 2 (1969) 65-71.

[2] B. Toby, EXPGUI, a graphical user interface for GSAS, J. Appl. Crystallogr. 34 (2001) 210-213.

[3] J.A. Barclay, W.A. Steyert, Materials for magnetic refrigeration between 2 K and 20 K, Cryogenics 22 (1982) 73-80.

[4] Z.W. Yang, J. Zhang, D. Lu, X. Zhang, H. Zhao, H. Cui, Y.-J. Zeng, Y. Long, Strong Magnetocaloric Coupling in Oxyorthosilicate with Dense Gd^{3+} Spins, Inorg. Chem. 62 (2023) 5282-5291.

[5] C. Wellm, J. Zeisner, A. Alfonsov, M.I. Sturza, G. Bastien, S. Gaß, S. Wurmehl, A.U.B. Wolter, B. Büchner, V. Kataev, Magnetic interactions in the tripod kagome antiferromagnet $Mg_2Gd_3Sb_3O_{14}$ probed by static magnetometry and high-field ESR spectroscopy, Phys. Rev. B 102 (2020) 214414.

[6] E.C. Koskelo, C. Liu, P. Mukherjee, N.D. Kelly, S.E. Dutton, Free-Spin Dominated Magnetocaloric Effect in Dense Gd^{3+} Double Perovskites, Chem. Mater. 34 (2022) 3440-3450.

[7] A. Zeleňáková, P. Hrubovčák, O. Kapusta, V. Zeleňák, V. Franco, Large magnetocaloric effect in fine Gd_2O_3 nanoparticles embedded in porous silica matrix, Appl. Phys. Lett. 109 (2016) 122412.

[8] V. Franco, A. Conde, Scaling laws for the magnetocaloric effect in second order phase transitions: From physics to applications for the characterization of materials, Int. J. Refrig. 33 (2010) 465-473.

[9] V. Franco, A. Conde, J.M. Romero-Enrique, J.S. Blázquez, A universal curve for the magnetocaloric effect: an analysis based on scaling relations, J. Phys.: Condens. Matter 20 (2008) 285207.

[10] J.Y. Law, V. Franco, L.M. Moreno-Ramirez, A. Conde, D.Y. Karpenkov, I. Radulov, K.P. Skokov, O. Gutfleisch, A quantitative criterion for determining the order of magnetic phase transitions using the magnetocaloric effect, Nat. Commun. 9 (2018) 2680.

[11] V. Franco, J.S. Blázquez, J.J. Ipus, J.Y. Law, L.M. Moreno-Ramírez, A. Conde, Magnetocaloric effect: From materials research to refrigeration devices, Prog. Mater. Sci. 93 (2018) 112-232.

[12] T.D. Shen, R.B. Schwarz, J.Y. Coulter, J.D. Thompson, Magnetocaloric effect in bulk amorphous $Pd_{40}Ni_{22.5}Fe_{17.5}P_{20}$ alloy, J. Appl. Phys. 91 (2002) 5240-5245.
